# Non-Invasive Human Embryo Metabolic Assessment as a Developmental Criterion

**DOI:** 10.3390/jcm9124094

**Published:** 2020-12-18

**Authors:** Marjan Motiei, Katerina Vaculikova, Andrea Cela, Katerina Tvrdonova, Reza Khalili, David Rumpik, Tatana Rumpikova, Zdenek Glatz, Tomas Saha

**Affiliations:** 1 Centre of Polymer Systems, Tomas Bata University in Zlin, Třída Tomáše Bati 5678, 76001 Zlin, Czech Republic; 2 Footwear Research Centre, University Institute, Tomas Bata University in Zlin, Nad Ovcirnou 3685, 76001 Zlin, Czech Republic; kvaculikova@utb.cz (K.V.); tsaha@utb.cz (T.S.); 3 Department of Biochemistry, Faculty of Science, Masaryk University, 62500 Brno, Czech Republic; 323512@mail.muni.cz (A.C.); glatz@chemi.muni.cz (Z.G.); 4 Department of Biology, Faculty of Sciences, University of Hradec Kralove, 50003 Hradec Kralove, Czech Republic; katerina.tvrdonova@ivfzlin.cz; 5 Department of Obstetrics and Gynecology, Faculty of Medicine, Masaryk, University Hospital Brno, 62500 Brno, Czech Republic; 6 Department of Paediatrics and Inherited Metabolic Disorders, First Faculty of Medicine, Charles University and General University Hospital in Prague, Ke Karlovu 2, 12808 Prague, Czech Republic; reza.khalili@lf1.cuni.cz; 7 Clinic of Reproductive Medicine and Gynaecology, 76001 Zlin, Czech Republic; david.rumpik@ivfzlin.cz (D.R.); tatana.rumpikova@ivfzlin.cz (T.R.)

**Keywords:** metabolomics, capillary electrophoresis, embryo development, culture media

## Abstract

The selection of a highly-viable single embryo in assisted reproductive technology requires an acceptable predictive method in order to reduce the multiple pregnancy rate and increase the success rate. In this study, the metabolomic profiling of growing and impaired embryos was assessed on the fifth day of fertilization using capillary electrophoresis in order to find a relationship between the profiling and embryo development, and then to provide a mechanistic insight into the appearance/depletion of the metabolites. This unique qualitative technique exhibited the appearance of most non-essential amino acids and lactate, and depleting the serine, alanyl-glutamine and pyruvate in such a manner that the embryos impaired in their development secreted a considerably higher level of lactate and consumed a significantly higher amount of alanyl-glutamine. The different significant ratios of metabolomic depletion/appearance between the embryos confirm their potential for the improvement of the prospective selection of the developed single embryos, and also suggest the fact that pyruvate and alanyl-glutamine are the most critical ATP suppliers on the fifth day of blastocyst development.

## 1. Introduction

Assisted reproductive technology (ART), despite epigenetic disruptions and low live birth rates, is still one hope of many subfertile couples. Although it is impossible to confirm an association between human ART and epigenetic disturbances [1], and these limited epigenetic variations are largely attenuated by adulthood [2], ART laboratories care about the quality control and quality assurance of ART policies and procedures [3]. In addition, many embryologists try to increase the pregnancy rate by the replacement of multiple embryos in a cycle. Several European countries disagree with this subject, owing to high obstetric, neonatal, and economic costs. Therefore, it is necessary to identify sensitive and predictive biomarkers for the differentiation of the embryos’ developmental potential. Recently, various criteria (i.e., cell number, morphological appearance, and embryo cleavage timing) have been utilized for embryo selection, which requires an extended embryo culture, time to monitor the embryo development, and an experienced embryologist. Due to the individual errors and detrimental effect of prolonged time on the human embryos [4], these criteria are relatively subjective and poor implantation predictors. Therefore, embryo selection requires a reliable predictive strategy, independent of or complementary to other predictors. 

Owing to the effect of exogenously-supplied substrates on the human preimplantation embryo, the consumption and secretion of amino acids (AAs) have the potential to be utilized for the selection of developmentally-competent single embryos, increasing the success rate and eliminating multiple births through in vitro fertilization (IVF) [5]. Another key aspect of preimplantation embryo development is carbohydrate metabolism, which is differentiated at distinct developmental stages. In the early stages of development, the tricarboxylic acid (TCA) cycle and its central substrates, pyruvate (Pyr) and lactate (Lac), are the main energy sources, which are replaced by glycolysis and increasingly glucose (Glc) consumption upon the transition from the morula to the blastocyst stage [6,7]. However, most knowledge on this subject has been derived mainly through the noninvasive separate assessment of the depletion/appearance of milieu components, such as AAs [8,9,10,11] and carbohydrates (i.e., Glc and its metabolites, Pyr and Lac) [6,12] in the early cleavage-stage embryos. For the establishment of a more reliable predictive biomarker, this study focused on the simultaneous evaluation of all these metabolites in the blastocyst stage of embryo development using a noninvasive, cost-effective, reproducible, rapid, and efficient strategy. 

More recently, the metabolites in the embryo’s milieu have been assessed by different methods, including near-infrared (NIR) [13,14], nuclear magnetic resonance (NMR) [15,16], high-performance liquid chromatography (HPLC) [5,10], gas chromatography (GC) [17,18] and capillary electrophoresis (CE) [6,9,11]. Among these techniques, NIR and NMR were rejected due to the increasing failure rate of the clinical trials, but chromatography was recognized as a promising method to distinguish the embryos with the potential to develop or arrest blastocysts in vitro. Thereafter, CE has been introduced as a promising separation technique with more significant potential due to its fast direct analysis, minimal sample volume, and high sensitivity [6]. 

Regarding the above-mentioned features, this study focused on the metabolic activity of growing and impaired embryos regarding the most important potential biomarkers. We aim to compare—safely and noninvasively—the appearance/depletion of AAs, Glc, Lac, and Pyr between single human embryos, which succeeded or failed to progress to the blastocyst stage during the fifth day post-insemination. According to Brison et al., who declared that the limitation of the length of time decreased the human embryo’s exposure to the detrimental conditions of the culture media [4], we utilized the fifth day of the embryo culture to distinguish the variability of the morphology, cell number, and viability of the blastocysts more accurately [12]. It is also noteworthy to distinguish the relationship between the embryo viability and metabolomics, and to provide a mechanistic insight into the release or consumption of metabolites from/by the embryos. 

## 2. Experimental Section

### 2.1. Chemicals and Reagents

All of the L-AAs, Glc, sodium L-Lac, sodium Pyr, boric acid, hydrochloric acid (HCl), (2-hydroxypropyl)-β-cyclodextrin, dextran sulfate sodium salt (from Leuconostoc spp. (DS, MW 500 kDa)), lithium hydroxide (LiOH) monohydrate, naphthalene-2,3-dicarboxaldehyde, picolinic acid, 94% polybrene (PB), sodium cyanide (NaCN), sodium deoxycholate, sodium dodecyl sulfate, sodium hydroxide (NaOH), sodium tetraborate decahydrate, and Tween^®^ 20 were purchased from Sigma-Aldrich (St. Louis, MO, USA). The acetonitrile and buffer solutions for the pH calibration were provided by Burdick & Jackson (Honeywell International, Muskegon, MI, USA) and Hamilton Bonaduz (Bonaduz, Switzerland), respectively. The deionized water (DI) (18.2 MΩ cm at 25 °C) was prepared using a Direct-Q^®^ 3 UV water purification system (Merck Millipore, Billerica, MA, USA). 

### 2.2. Samples of Human Embryo Culture Medium

In total, 58 patients with an average age of 25–35 years undergoing ART were treated according to the standard protocols, and gave informed consent for their participation in this clinical research, which was in accordance with the ethical standards of the institutional and national research committee, and with the 1964 Helsinki declaration and its later amendments (or comparable ethical standards). Hormone therapy (i.e., FSH, LH and an antagonist of GnRH) was performed in order to stimulate ovarian follicular growth. When at least three follicles reached a mean diameter of 18 mm, Ovitrelle^®^ 6500 IU was administered in order to induce ovulation. In total, 36 h after the Ovitrelle^®^ administration, oocyte retrieval was carried out transvaginally by the use of ultrasound technology to guide a needle into each ovary. After removing the fluid from the follicles and intracytoplasmic sperm injection/physiological intracytoplasmic sperm injection (ICSI/PICSI) fertilization, the fertilized oocytes from each patient were cultured in a 4-well dish (Nunc, Thermo Fisher Scientific, Denmark) containing 500 μL fertilization medium (Cook, Australia) in the presence of 6% CO_2_ at 37 °C. After 18 h, a single fresh embryo was randomly transferred to the 25 μL single-stage G-TL™ culture medium (Vitrolife, Sweden) under Sydney IVF Culture Oil (Cook, Australia) in an EmbryoSlide culture dish (Vitrolife A/S Denmark) and placed into an EmbryoScope time-lapse incubator (Vitrolife, Sweden) at 37 °C in an atmosphere of 6% CO_2_ for 5 days. On the fifth day, the embryo culture media containing the resultant blastocysts (Grade 1 and 4), and the free culture media were aspirated using a 25 μL Hamilton microsyringe equipped with a blunt tip. The media were transferred to a 1 mL glass vial with a plastic cap containing a 100 μL glass insert with plastic feet. Between each sampling, the Hamilton microsyringe was washed using a flushing solution (1:1 ultra-pure water/methanol solution). Finally, the culture media were categorized into three groups (i.e., free culture, growing, and impaired embryo media) and were shipped from the clinic on dry ice, stored in a refrigerator at −20 °C, and analyzed within 2 months.

### 2.3. General Sample Preparation and Metabolite Determination Using CE

After thawing the samples at room temperature (RT), paraffin oil removal and protein precipitation were carried out. The paraffin oil was removed by three-step centrifugation at 10,000× *g* in the microcentrifuge Minispin^®^ plus (Eppendorf, Hamburg, Germany), firstly for 5 min to form a phase interface between the oil and the medium, then 10 µL of the lower phase for the next 5 min, and finally, 5 µL of the lower phase for the last 5 min. In total, 8 µL acetonitrile was mixed with 2 µL of the lower phase, thoroughly stirred, and kept for 5 min at RT to precipitate. The precipitate was separated from the liquid by centrifugation at 10,000× *g* for 5 min. The metabolite analysis was performed on 5 µL of the collected supernatants after derivatization in the case of the AAs [11], or dilution in the case of Pyr, Lac [6], and Glc [19].

### 2.4. Determination of Amino Acids (AAs) Using Capillary Electrophoresis—Light Emitting Diode Fluorescence Detection (CE-LIF)

The derivatization and analysis of the AAs were performed according to the procedure described in detail in a previously published paper [11]. Briefly, 15 µL 8.33 mM sodium cyanide, 25 µL reaction buffer (200 mM boric acid/NaOH, pH 9.0) and 5 µL 25 mM naphthalene-2,3-dicarboxaldehyde were added to 5 µL of the collected supernatant. The derivatization was conducted for 45 min at 25 °C, and 650 rpm, and the reaction was ended by freezing at −70 °C. The derivatized samples were stored in a freezer, and each sample was thawed just before its analysis. 

The determination of the derivatized AAs was performed using an Agilent G7100A CE System (Agilent Technologies, Waldbronn, Germany) coupled with an external collinear LEDIF detector ZETALIF™ LED 480 (Adelis, Labège, France; 488/515 nm excitation/emission wavelength). The separation was performed in bare fused-silica capillaries (Polymicro Technologies, Phoenix, AZ, USA) of 50 µm inner diameter (I.D.), 375 µm outer diameter (O.D.), 66.0 cm total length, and 45.0 cm effective length. The capillary was maintained at 25 °C, and the separation voltage was +30 kV, with an initial voltage rise of 2.5 kV s^−1^. The derivatized sample was injected into the capillary with a pressure of 50 mbar (5 kPa) for 3 s. The background electrolyte (BGE)—composed of 50 mM sodium tetraborate, 73 mM sodium dodecyl sulfate, 5 mM sodium deoxycholate, and 2.5 mM (2-hydroxypropyl)-β-cyclodextrin (pH ≈ 9.3)—was prepared weekly and stored at laboratory temperature. Before each analysis, the capillary was rinsed with BGE for 2 min. After each analysis, the capillary was rinsed with DI water, 1% (*w*/*w*) Tween^®^ 20, and DI for 0.5, 2, and 1 min, respectively. Calibration curves were constructed within the expected concentration range of each AA in the G-TL™ medium. The migration time and peak area repeatability were below 0.5% and 4.3%, respectively. The intermediate precision was the same for the migration times, but below 9.9% for the peak areas. The limits of detection ranged from 12.6 nM (His) to 39.3 nM (Tau).

### 2.5. Determination of Pyr and Lac Using a Capacitively-Coupled Contactless Conductivity Detector (CE-C^4^D)

The determination of Pyr and Lac was performed using an Agilent G7100A CE System coupled with in-house assembled CE-C^4^D (CCD-Admet, Rev. 5.06; 1,0 MHz, T) based on a previously-published paper [6], with a revision of the BGE composition in order to allow the simplification of the sample preparation. To the 5 µL of collected supernatant was added to 95 µL of DI, resulting in a 100× diluted sample of the spent culture medium. Bare fused-silica capillaries of 50 µm I.D. and 375 µm O.D. were purchased from Polymicro Technologies. Each newly-cut capillary with a total length of 64.0 cm and an effective length of 49.5 cm was rinsed at 50 °C with DI, 1 M LiOH, 0.1 M LiOH, and DI water for 5, 20, 10 and 10 min, respectively. The inner capillary wall was then modified using a dynamic coating of charged polymeric layers (i.e., polybrene (PB) and dextran sulphate (DS)) at 25 °C, as follows. The capillary was sequentially rinsed with DI, 5% (*w*/*v*) PB, DI, 3% (*w*/*v*) DS, DI, 5% (*w*/*v*) PB, and DI for 5, 20, 5, 30, 10, 20, and 5 min, respectively. Finally, the capillary was rinsed with BGE for 60 min, and the BGE was left overnight in the capillary. 

The BGE was prepared freshly by dissolving the appropriate weight of picolinic acid in 80% of the final volume of DI and adjusting the pH to 4.7 using 1 M LiOH. The solution was quantitatively transferred to a volumetric flask and filled with DI in order to obtain 20 mM picolinic acid/LiOH, with a pH of 4.7. The solution was thoroughly mixed, filtered using a 0.45 µm Nylon membrane filter (Fisher Scientific, Pardubice, Czech Republic), and degassed in a Bransonic^®^ 1510E DTH ultrasonic bath (Branson Ultrasonic, Danbury, CT, USA) for 5 min. The capillary was maintained at 25 °C, and the separation voltage was −24 kV, with an initial voltage rise of 2.5 kV s^−1^. The 100× diluted sample was injected into the capillary with a pressure of 50 mbar (5 kPa) for 15 s. Before each analysis, the capillary was rinsed with 0.5% (*w*/*v*) PB and BGE for 3 and 5 min, respectively. After each analysis, the capillary was rinsed with DI for 2 min. Calibration curves were constructed within the expected concentration range of Pyr (0.5–12.5 µM) and Lac (10–250 µM) in the G-TL™ medium. The migration time repeatability for the Pyr and Lac was 0.5% and 0.6%, respectively. The peak area repeatability for the Pyr and Lac was 3.1% and 3.3%, respectively. The intermediate precision of the migration time for the Pyr and Lac was 0.8% and 0.9%, respectively. The intermediate precision of peak area for the Pyr and Lac were and 6.7% and 5.0%, respectively. The achieved limits of detection for the Pyr and Lac were 0.08 µM and 0.34 µM, respectively.

### 2.6. Determination of Glc Using CE-C^4^D

The determination of the Glc was performed using an Agilent G7100A CE System coupled with an in-house assembled C^4^D (‘CD-Admet’, Rev. 5.06; 1.0 MHz, T) based on a previously-published paper [19], with optimization of the BGE concentration and sample injection in order to fit the employed C^4^D and meet the sensitivity demands for Glc determination in the culture medium. Briefly, bare fused-silica capillaries of 10 µm I.D. and 375 µm O.D. were purchased from Polymicro Technologies. Each newly-cut capillary with a total length of 33.0 cm and an effective length of 18.5 cm was rinsed at 50 °C with DI, 1 M NaOH, 0.1 M NaOH, and DI for 10, 40, 20, and 20 min, respectively. The BGE composed of 40 mM NaOH (pH ≈ 12.5) was prepared weekly and stored at 4 °C. The capillary was maintained at 25 °C, and the separation voltage was +10 kV, with an initial voltage rise of 2.5 kV s^−1^. The 50× diluted sample with DI was injected into the capillary with a pressure of 50 mbar (5 kPa) for 120 s. Before each analysis, the capillary was rinsed with BGE for 5 min. At the end of the day, the capillary was thoroughly rinsed with DI, 0.1 M HCl, and DI for 15, 60, and 60 min, respectively. The calibration curve was constructed within the expected concentration range of Glc (2–50 µM) in the G-TL™ medium. The migration time and peak area repeatability for Glc were 3.0% and 2.3%, respectively. The intermediate precision of the migration time and peak area for Glc were 3.8% and 3.1%, respectively. The estimated limit of detection for the Glc was 0.46 µM.

### 2.7. Statistical Analysis

The data acquisition and peak integration were performed using the Agilent ChemStation software Rev. C.01.05 (Agilent Technologies, Waldbronn, Germany). The linear regression was performed using Microsoft Office Excel 2010 (Microsoft Corporation, Albuquerque, NM, USA). All of the data were tested for normality using the Shapiro–Wilk test (*p* > 0.05) [20] and the visual inspection of the histograms, normal Q–Q plots, and box plots, which demonstrated the approximately normal distribution of the variables. Thereafter, the differences among the groups were ascertained using multivariate analysis of the variance (MANOVA), and were presented as mean ± standard deviation (SD). In order to further explore the correlations among the observed variables, a principal component analysis (PCA) was also performed.

## 3. Results

The metabolic profiling was performed on three different media, including the free culture media and the media containing single human embryos, which grew to the blastocyst stage and were arrested before the formation of the blastocysts, with an average grade of 1 and 4, respectively. These average grades refer to the appearance of the cells in the embryos. Herein, the embryo grade 1 was related to the embryos containing cells with the same size and no fragmentation, and grade 4 was attributed to the equal or unequal cells with moderate to heavy fragmentation. The metabolite profiles of these three groups are summarized in Table 1, and the net appearance/depletion of each metabolite was calculated with reference to the mean of the control droplets cultured alongside them.

### 3.1. Appearance/Depletion of AAs by Embryos on the Fifth Day of Fertilization

**Figure 1:**
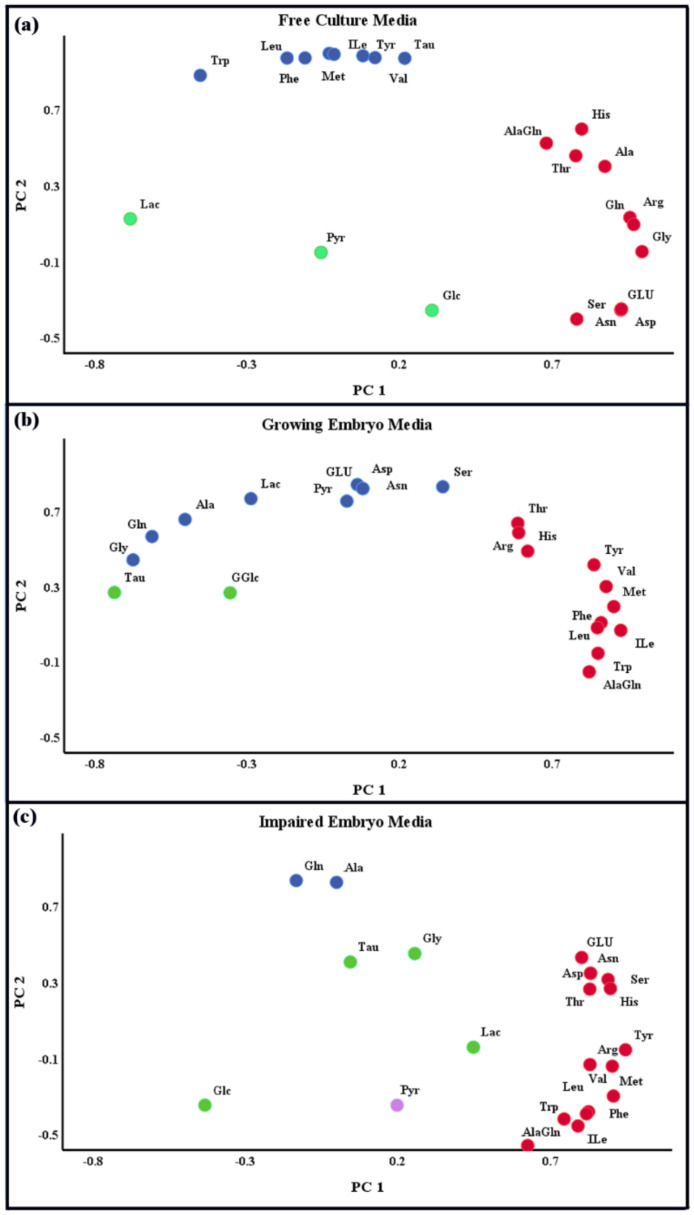
PC analysis of the free culture media (**a**), and the media containing the growing (**b**) and impaired embryos (**c**). Each circle represents different metabolites, wherein the color indicates their relativity. In the free culture media, the essential/conditionally-essential AAs, non-essential AAs, and carbohydrates are in separate groups with strong correlations. Nonetheless, this order is disturbed in the other two media, especially for the impaired embryos, when most of the AAs are correlated together.

**Figure 2:**
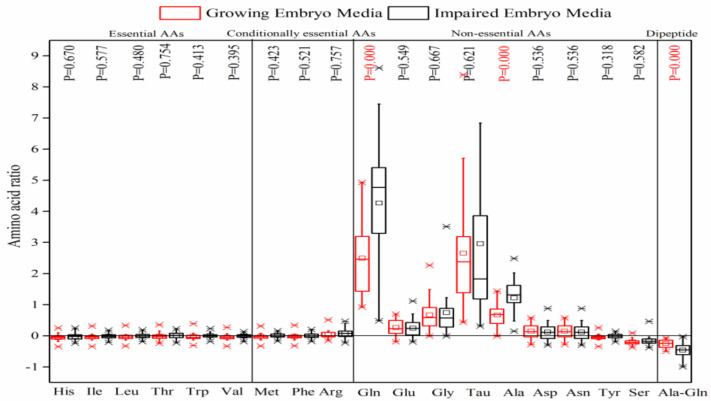
Comparison of the depletion/appearance of AAs by the growing and impaired human embryo media on day 5. This demonstrates the appearance of most non-essential AAs except Ser and the dipeptide of Ala-Gln in a manner that impaired the embryos, which secreted considerably higher levels of Gln and Ala, and consumed a significantly higher amount of Ala-Gln. Significant values have been marked in red above the graph (*p* value < 0.05).

The mean concentration of AAs and Ala-Gln dipeptide in the free culture media and the media containing the growing and impaired embryos to implant was shown in Table 1. It was interesting that the Ala-Gln concentration decreased significantly in the media containing the growing (121.0 ± 20.6 µM) and impaired embryos (87.0 ± 34.5 µM) against the blank (161.1 ± 11.2 µM). The results regarding non-essential AAs demonstrated a considerable decrease of Ser concentration, and a significant increase of Ala, Gln, Glu, Gly, and Tau concentrations (*p* value < 0.05) in the media for both embryos, but the considerable difference between the two kinds of embryos was distinguished only in the mean concentration of Ala and Gln (*p* < 0.05). The strong correlation of Ala and Gln was clearly observed in the impaired embryo media (Figure 1C). The concentration of the other AAs exhibited no significant difference among the free culture media, and the media containing the growing and impaired embryos to implant. Figure 2 also confirmed that there were neither depleted nor significant differences between the growing and impaired embryos in the consumption of most essential and conditionally-essential AAs. However, most non-essential AAs—except Tyr and Ser—were released to the culture media; the appearance of Ala and Gln increased significantly in the impaired embryos (*p* < 0.05). The PC analysis also confirmed that the non-essential AAs underwent greater correlation coefficients in the growing embryo media as opposed to the impaired media (Figure 1).

### 3.2. Appearance/Depletion of Carbohydrates by Embryos on the Fifth Day of Fertilization

**Figure 3:**
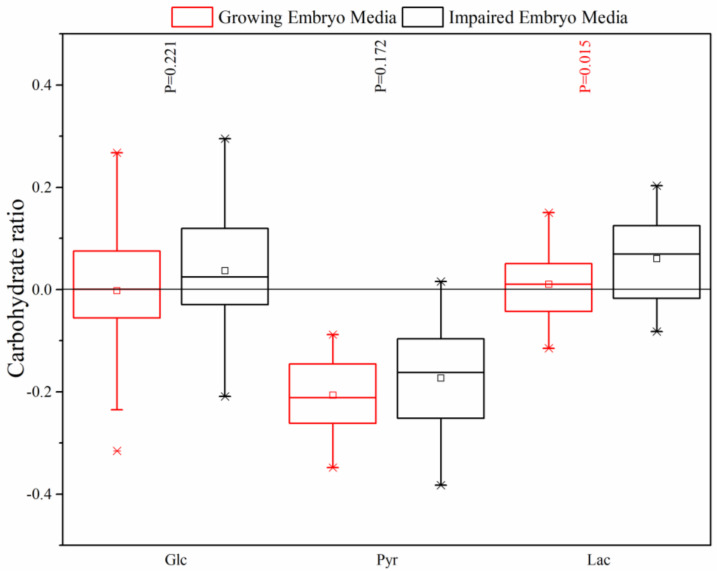
Comparison of the depletion/appearance of Glc, Pyr and Lac by the growing and impaired human embryos on day 5. The inset shows that Lac was significantly different between the growing and impaired embryos. Significant values have been marked in red above the graph (*p* value < 0.05).

According to Table 1, there were no significant changes in the Glc tolerance of the free media (910.0 ± 28.85 µM) or the media containing the growing (907.9 ± 114.8 µM) and impaired (943.4 ± 106.8 µM) embryos on day 5. Nonetheless, Pyr demonstrated a statistically significant lower concentration in the growing (286.5 ± 25.1 µM) and impaired embryos (298.6 ± 37.7 µM) against the free media (361.2 ± 21.2 µM). However, there was no significant difference between the mean concentration of Pyr in the growing and impaired embryos (*p* > 0.05). Table 1 also demonstrates that the mean Lac concentration for the growing embryos (8732.4 ± 592.6 µM) did not change significantly against the free media (8644.6 ± 166.2 µM), but the impaired embryos showed a considerable increase of Lac concentration (9163.9 ± 711.1 µM). Figure 3 confirms that the growing and impaired embryos did not show a considerable appearance/depletion of Glc in the culture media, and there was no significant difference in the amount of consumed Glc between the growing and impaired embryos (*p* = 0.221) either. However, there was a little release in the impaired embryos, which is not significant. Although Pyr disappearance markedly increased in the culture media by the growing (*p* < 0.05) and impaired embryos (*p* < 0.05); Lac was released considerably by the impaired embryos in comparison to the growing embryos (*p* = 0.015). The PC analysis also demonstrated that there was a strong correlation between Pyr and Lac in the growing embryo media, which was replaced with Glc and Lac in the impaired embryo media. Considering these temporal correlations among the metabolites, the media beyond the PCA path might correspond to anomalous embryonic developments, and perhaps dead embryos (Figure 1).

## 4. Discussion

Regarding the studies on embryo viability [6,9,10,11], the utilization of exogenous substrates and their noninvasive turnover assessment can give a comprehensive picture of embryo metabolism. Exogenous AAs (i.e., the inclusion of all essential, conditionally-essential, and non-essential AAs in addition to dipeptide of Ala-*Gln*) and carbohydrates (Glc, Pyr and Lac), among other supplements, are utilized widely in the culture media due to their potential benefits for the embryonic development [21]. However, the development and differentiation of the embryos relies on intracellular AAs, the exogenous supplies are specifically incorporated into polypeptide structures during the early stages of embryonic development, and the total polypeptide content will be constant until the blastocyst stage [8]. In mouse embryo development, non-essential AAs are particularly recommended for early cleavage stage development, and a mixture of all of the AAs is the best combination to increase blastocyst formation (8-cell to blastocyst stages) [5]. In addition to being protein precursors, they act as embryo metabolism regulators, external paracrine signaling molecules, osmolytes, and buffers for the adjustment of the intracellular pH [22].

On this basis, an alternative method was used to detect the differences in the AA turnover between the viable and non-viable embryos on day 5 post-insemination. Due to an 8-cell embryo requirement to a mixture of essential [5] and conditionally-essential AAs [8] for transformation to blastocyst, it was expected that these AAs would be depleted notably in the culture media on day 5. Nonetheless, this study demonstrated that they were neither depleted, nor was there a significant difference between the growing and impaired embryos in the consumption of these AAs.

Female reproductive tract fluids contain high concentrations of certain non-essential AAs, such as Gln, Tau, Glu, and Gly, which confirm their important roles in preimplantation embryo development [23]. Herein, the most striking data were provided by Ala-Gln dipeptide and non-essential AAs (especially Gln, Tau, Ala, Gly) which demonstrated the net depletion and appearance in the culture media throughout embryo development, respectively. Ala-Gln dipeptide is introduced to all ART culture media as a standard practice because of its higher stability than individual Gln degraded to ammonium spontaneously and metabolically [24]. Although Ala-Gln provides osmoregulation with lower efficacy than individual AAs, it can efficiently reduce the magnified ammonium accumulation in preimplantation embryo culture media [24,25]. The dipeptide may be uptaken within the cells by a specialized dipeptide transporter or an AA transporter after its hydrolyzation into its constituent AAs by secreted or membrane-bound peptidases [26]. The depletion of Ala-Gln and the appearance of the constituent AAs suggests the pivotal role of peptidases that hydrolyze Ala-Gln into Ala and Gln outside the cell. The significantly higher consumption of Ala-Gln and production of Ala and Gln, especially by impaired embryos, can also be discussed in terms of their greater metabolic activeness [5,10]. The significantly high release of Ala and Gln is consistent with Houghton et al. [5], and inconsistent with Sturmey et al., who declared that mouse embryos at blastocyst stages consumed Gln at a significantly higher rate than the early cleavage stages [8]. L-Gln is considered to be an essential component of culture media during embryo development over the 2- to 8-cell stages in a number of species [24,27]. It can be metabolized to other AAs (i.e., Glu, Ala, and Asp) [28] and ATP through the TCA cycle, synthesizes de-novo purine and pyrimidine, protects the embryo against oxidative stress, and acts as an organic osmolyte and a putative regulator in Glc metabolism [8]. Ala also provides a means of disposing of toxic ionic ammonia (NH_4_^+^) via transamination with Pyr [5,8].

Tau was another highly-released AA in the culture media by both the growing and impaired embryos, with no significant difference on day 5 post-insemination. Tau is the most plentiful non-protein AA in female reproductive tract fluids and male semen, with diverse cytoprotective activity against oxidative damage and Glu-induced excitotoxicity [23]. Tau functions as a free radical scavenger [8], an intracellular organic osmolyte, and a membrane stabilizer by modulating the intracellular calcium homeostasis. Although little is known about the effect of Tau on human preimplantation embryo development, this semi-essential nutrient seems to support the developmental stages of the 2–4-cell blastocyst [23].

Gly, a critical AA for the preimplantation development of both early and late-stage embryos [29] was also released to the media. Gly is abundant in oviduct fluid [29], and has been reported to be an essential precursor of polypeptide and nucleic acid synthesis, one of the key organic osmolytes and heavy metal chelators, which undergoes less apoptosis in Gly-cultured embryos [8,27,30,31]. Tracking the fate of orally administered ^15^N-Gly showed that the Gly nitrogen group is mainly transferred to Ser, urea, Gln/Glu, Ala, and other AAs (Leu, Ile, Val, Orn, Pro, and Met) [32]. The most likely explanation is that the consumption of Ser and the release of Gly in the culture media can be attributed to the de novo synthesis of Gly by Ser, and the inter-convertibility of these AAs [31].

Asp and Asn are the other non-essential AAs that showed a less apparent release in the media, which can be discussed with regard to the key role of Asp in energy metabolism by the Mal–Asp shuttle. However, the studies confirmed that the Mal–Asp shuttle controls carbohydrate utilization in vivo and in vitro by affecting the Glc and Lac metabolism of the early cleave stage embryos, but not the blastocysts [33]. According to Uyar et al., Pyr and Lac are the main energy sources in the early stages of development, while Glc consumption predominates at the blastocyst stage in several species [7]. However, our results demonstrated a markedly high Pyr uptake, Lac release, and no change in the Glc tolerance at the blastocyst stage on day 5. The preferential consumption of Pyr in this stage can be explained by the significant Lac production and Glc uptake reduction due to the inadequate activity of Mal–Asp shuttle, which is necessary for Lac and Glc metabolism [33]. Conaghan et al., also confirmed that the accumulation of Pyr significantly decreased in the presence of a high Lac concentration between day 2 and day 6 [34]. It can be concluded that, in this stage, the balance between mitochondrial and cytoplasmic respirations has been altered, and Pyr is the most important source producing a usable form of cellular energy, ATP, through the TCA cycle and oxidative phosphorylation [8]. The higher mean Lac release in the impaired embryos, in comparison with growing embryos, could otherwise indicate the disruption of the mitochondrial and cytoplasmic metabolisms, maintained by the Mal–Asp shuttle. Mitchell et al. also confirmed that the fully developed mouse blastocyst with subsequent viable pregnancy was closely linked to the maintenance of the balance [33]. Our findings are in contrast with Gardner et al., who demonstrated that growing embryos for implantation consumed greater quantities of Pyr and Glc than embryos which failed to reach the blastocyst development stage on day 4 post-insemination [12]. Conaghan et al. also declared that the consumption of Glc and the production of Lac was higher for growing embryos than impaired embryos due to the presence of uncoupled mitochondria with highly-permeable inner membranes to protons [34].

**Figure 4:**
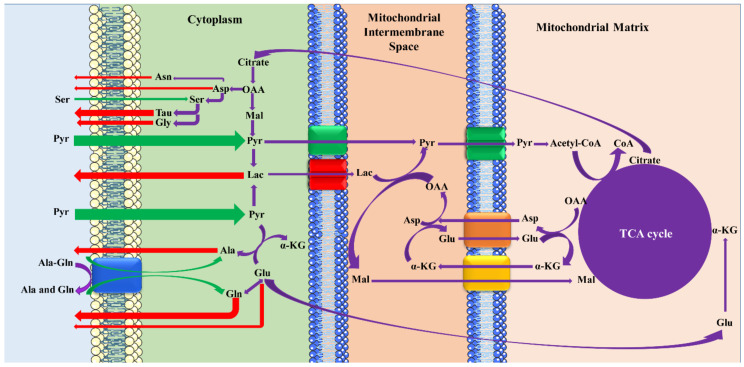
Summary of the proposed mechanism among the metabolites on the fifth day of embryo development. Pyr and Lac transfer through H^+^-monocarboxylate cotransporters. Ala-Gln may be uptaken within the cells by the AA transporter after hydrolyzation by peptidase into its constituents. Entrance, exit and intercellular interactions of different metabolites have been illustrated in green, red and violet arrows, respectively. TCA: tricarboxylic acid, OAA: oxaloacetic acid, α-KG: α-ketoglutarate, Mal: malate.

The proposed scheme of the mechanisms among the metabolites on the fifth day of embryo development is presented in Figure 4. The cationic, zwitterionic [35], and anionic AAs [36] are transported by Na^+^-independent, Na^+^-dependent/independent, and Na^+^-dependent/independent processes, respectively. After Pyr’s entrance into the embryos through H^+^-monocarboxylate cotransporters [37], it greatly affects the production of the most critical energy source, ATP, and also the AA profile in the blastocysts’ development. Under the inadequate activation of the Mal–Asp shuttle, blastocysts consume appreciable quantities of Pyr and produce large amounts of Lac, especially by impaired embryos, which can be attributed to the competitive transport of carboxylic acids [38]. According to the results, the transfer of the dipeptide, Ala-Gln, is accompanied by hydrolysis to constituents such as Gln, which is metabolized to Glu, and then affects the ATP production by the TCA cycle and AA profiles. Therefore, the consumption of Ala-Gln and its indirect effect on the production of AAs may influence their release. For example, both exogenous and endogenous Ser is metabolized to Tau and Gly, which lead to the disappearance of Ser and the high appearance of Tau and Gly in the culture media.

## 5. Conclusions

Our current findings suggest that CE can be a unique technique in this respect, and the measurement of the depletion/appearance of the metabolites of individual human embryos provides functional insights into the biological procedures attributed to the acquisition of embryo developmental competence in vivo and following assisted conception. However, the overall metabolite depletion/appearance may vary qualitatively among different studies, which can be associated with the stochastic variability of the measurement techniques, the composition of the culture media, the embryonic development stages, and the fact that all blastocysts are not viable in the culture media. Finally, it is often appropriate to take a broader perspective in a meta-analysis than a single clinical trial in order to obtain applicable criteria and standards relating to this subject.

## Figures and Tables

**Table 1:** Mean concentration of metabolites (µM) in the free culture media and the media containing the growing and impaired single embryos to implant.

Metabolites		Free Culture Media (µM)	Growing Embryo Media (µM)	Impaired Embryo Media (µM)
Essential AAs	His	81.2 ± 9.4	77.7 ± 7.5	78.6 ± 8.7
	Ile	196.9 ± 18.9	191.8 ± 19.6	194.6 ± 17.1
	Leu	199.5 ± 17.5	195.5 ± 20.6	199.2 ± 17.8
	Thr	159.2 ± 16.9	157.8 ± 17.6	159.3 ± 18.5
	Val	187.9 ± 16.2	182.6 ± 17.0	186.2 ± 14.5
	Trp	23.7 ± 2.7	23.4 ± 2.8	23.9 ± 2.3
Conditionally essential AAs	Phe	99.0 ± 8.4	97.9 ± 10.5	99.6 ± 9.1
	Met	49.6 ± 4.3	48.9 ± 4.7	49.9 ± 4.0
	Arg	174.2 ± 21.7	183.2 ± 27.1	185.4 ± 26.4
Non-Essential AAs	Ala	79.4 ± 13.3 ^a^	134.27 ± 24.4 ^b^	177.9 ± 42.8 ^c^
	Asp	39.5 ± 13.3	45.0 ± 9.7	43.4 ± 8.5
	Glu	32.9 ± 9.8 ^a^	41.4 ± 8.9 ^b^	40.0 ± 8.0 ^b^
	Gly	179.2 ± 26.8 ^a^	302.4 ± 90.3 ^b^	315.5 ± 133.5 ^b^
	Asn	39.5 ± 13.2	45.0 ± 9.7	43.4 ± 8.8
	Gln	15.8 ± 2.3 ^a^	56.2 ± 16.5 ^b^	84.1 ± 30.5 ^c^
	Ser	88.3 ± 36.4 ^a^	71.7 ± 8.7 ^b^	74.1 ± 13.9 ^b^
	Tyr	90.8 ± 7.5	87.6 ± 7.9	89.8 ± 7.6
	Tau	44.1 ± 3.3 ^a^	162.9 ± 82.2 ^b^	177.4 ± 130.4 ^b^
Dipeptide	Ala-Gln	161.1 ± 11.2 ^a^	121.0 ± 20.6 ^b^	87.0 ± 34.5 ^c^
Carbohydrates	Glc	910.0 ± 28.85	907.9 ± 114.8	943.4 ± 106.8
	Pyr	361.2 ± 21.2 ^a^	286.5 ± 25.1 ^b^	298.6 ± 37.7 ^b^
	Lac	8644.6 ± 166.2 ^a^	8732.4 ± 592.6 ^a^	9163.9 ± 711.1 ^b^

Values in each row marked with different letters in superscript denote a significant difference between the groups (*p* value < 0.05).
